# An examination of the quality of kidney stone information on YouTube and TikTok

**DOI:** 10.1007/s00240-025-01713-4

**Published:** 2025-02-25

**Authors:** Zuzanna Augustyn, Helen L Richards, Alice McGrath, Yvonne Swanton, Derek B Hennessey

**Affiliations:** 1 School of Medicine, University College Cork, Cork, Ireland; 2 Department of Urology, Mercy University Hospital, Cork, Ireland; 3 Department of Clinical Psychology, Mercy University Hospital, Cork, Ireland; 4 School of Food and Nutritional Sciences, University College Cork, Cork, Ireland

**Keywords:** Urolithiasis, Education, Social media, Quality

## Abstract

Social media is increasingly consulted by patients for health-related information. The quality of this information is unknown and unregulated. This study aimed to investigate the quality of kidney stones information on two popular video platforms, YouTube and TikTok, and examine factors related to its quality. A search using keywords of “kidney stones” alongside “information, prevention, treatment” was used across YouTube and TikTok. The top 50 videos on each platform were rated by 4 independent reviewers on: general information, epidemiology, symptoms/presentation, treatment, and prevention. The American Urological Association’s kidney stone curriculum was utilised as the benchmark for quality. The total number of views across the 100 videos was over 46 million. Overall, 91% of videos were educational in terms of genre. Misinformation was present in 34% of TikTok and 2% of YouTube videos. YouTube received significantly higher quality ratings across all parameters bar prevention (z’s>-4.79 p’s < 0.001); however, less than 50% of YouTube videos were rated as good quality. There was no association between quality and the number of likes or views across platforms (p’s > 0.3). The quality of information presented across platforms was very variable with over half of videos considered poor quality. Given the high usage of social media as a source of health information it is important that healthcare providers and the general public are aware of the limits of information available on both platforms and continued efforts are made to develop high quality videos suitable for patient consumption.

**Supplementary Information:**

The online version contains supplementary material available at 10.1007/s00240-025-01713-4.

## Introduction

Online websites and social media platforms (SoMe) are increasingly recognised as one of the most popular options for patients seeking information regarding their health conditions [1]. Among SoMe platforms, YouTube and TikTok are the largest, with in the region of 2.2 and 1 billion monthly active users, respectively. A recent study of YouTube users [2] showed up to 88% of individuals seeking health information online and a further 44% making decisions to consult doctors based on online content. Given the volume of users of SoMe, the content and accuracy of information posted is imperative. While some studies have suggested the positive benefits of YouTube content in relation to health-related decisions [3] others have highlighted caution over accuracy of online content [2]. Despite there being misinformation policies in place on both platforms, and a specific medical misinformation policy for YouTube there are no prerequisites for uploading health related and medical content on YouTube and TikTok. Thus, the potential for individuals to access misinformation via these SoMe platforms is a concern and has suggested to influence the risk of negative health outcomes at both an individual and societal level [4]. Although medical misinformation is a topic commonly discussed in the media, it may be difficult for the general public to discern which online resources they can trust [5], especially with the exponential rise of short-form content, as seen on platforms like TikTok, and the overabundance of information presented to platform users.

The increasing prevalence of kidney stones (KS) in the population [6, 7] creates a growing audience for kidney stone information consumption on YouTube and TikTok. Alongside this the volume of KS content may be further amplified due to the fact that: there are often attempts to treat KS at home through non-medical interventions and home remedies [8, 9]; KS are a condition which have suboptimal patient education resources [10]; and there are variable levels of health literacy observed in individuals with KS [10, 11].

Studies related to online information about KS are limited. One study, evaluated the content of TikTok content relating specifically to KS surgery [12], and a further study, now over 10 years old, examined the usefulness of information about KS on YouTube videos and reported that 58% of KS videos on YouTube had useful information and almost one fifth of videos were misleading [13]. The accuracy and quality of KS information contained across available SoMe content has not to our knowledge been previously evaluated.

The objective of the current study was to examine the most frequently viewed KS content on YouTube and TikTok and to appraise the quality of the KS information against agreed standards of the American Urological Association’s curriculum [14]. We also aimed to compare the two video platforms in terms of quality of information provision and misinformation.

## Methods

### Ethical approval

was obtained from University College Cork’s social research ethics committee. All data gathered is freely available on the respective platforms in accordance with the platforms’ terms of service.

### Data source & study population

Searches for videos were conducted on 20/03/2024 using the term ‘kidney stones’ along with the related terms ‘information, prevention, treatment’. We searched YouTube and TikTok respectively and new accounts were created on each platform to avoid affecting the results through personalised algorithms.

The results were sorted by the default setting of ‘relevance’ on YouTube and ‘top’ on TikTok. The first 50 videos were selected for each platform to be reviewed giving a total sample of 100 videos. Videos classified as YouTube ‘shorts’ (the platform’s short-form vertical content, different from regular YouTube videos), and videos on either platform not available in English were not included in the sample. The searches were not updated beyond 20/03/2024.

## Data extraction

Data was extracted from each YouTube or TikTok video which included the date of upload, the genre (educational, personal experience, comedic/entertainment, ‘how to’, advertisements) and the number of likes, views, and comments at the time of sample collection. The qualifications or accreditations of the video uploader/presenters were also recorded where available.

## Procedure

Four reviewers independently rated each of the 100 videos. The reviewers consisted of: a consultant urologist, a urology trainee, a medical student, and a non-clinical research team member. Each video was reviewed individually and rated for quality in 5 categories; general information, epidemiology, presentation/symptoms, treatment, and prevention. Five-point Likert scales ranging from ‘very poor quality’ to ‘very good quality’ were utilised to rate the quality of the videos under each of the categories. A ‘non-applicable’ option was used when a category was omitted from a video. Incidences of misinformation identified by the reviewers were noted in qualitative form. The expert reviewers (Consultant Urologist and Urology trainee) used the American Urological Association’s kidney stone curriculum [14] as the ‘gold standard’ of information quality. The non-expert reviewers (medical student and non-clinical team member) were instructed to rate the videos based on their own knowledge of the topics in the videos. All data collected from the videos and ratings from the 4 reviewers were entered onto an SPSS spreadsheet.

## Analysis

Data was entered onto SPSS 29. Descriptive statistics (medians, percentages) were used to summarise the data in terms of number of views, likes and comments for each video. Kendall’s W was used to examine agreement between the 4 raters in terms of video quality. Differences between the 2 video platforms were examined with Mann-Whitney U test and associations between variables (e.g. quality of video and number of likes or views) was examined with Spearman rank correlation coefficient. Given the number of tests undertaken and to reduce the likelihood of type 1 error significance levels were set to *p* < 0.01.

## Results

The total number of views across the 100 videos was over 46 million. The median number of views was 125k on YouTube and 31k on TikTok. The median number of likes was 1200 on YouTube and 552 on TikTok. See Table 1 for further descriptive data relating to the video content.

**Table 1:** Descriptive data related to ‘views’, ‘likes’, ‘qualification of uploader’, ‘genre’ and misinformation of videos viewed on YouTube and TikTok

Variable	YouTube *n* = 50	TikTok *n* = 50
Total views median (IQR range)	125,000 (27631–769913)	31,000 (2152–82775)
Total likes median (IQR range)	1200 (232–4750)	552 (49-2244)
Total comments median (range)	40 (0-282)	22 (2–82)
Qualification of uploader n (%)		
*Healthcare professional* *Hospital/Clinic/Journal* *Other* *Qualification not disclosed*	11 (22) 34 (68) 0 5 (10)	29 (58) 0 3 (6) 18 (36)
Genre n (%)		
*Educational* *Tutorial* *Entertainment/comedy* *Personal experience* *Advertising/promotional*	49 (98) 1 (2) 0 0 0	42 (84) 2 (4) 1 (2) 2 (4) 9 (18)
Misinformation n (%)	1 (2)	17 (34)

90% of YouTube videos were presented/uploaded by healthcare professionals or an accredited hospital/clinic/journal, whilst for the remaining 10% of videos the information was unavailable. On TikTok 58% of presenters/uploaders were healthcare professionals. The most common genre of video on both platforms was ‘educational’, 98% on YouTube and 84% on TikTok. There was a wider variety of genres on TikTok than YouTube.

Of the 50 TikTok videos 18% contained product advertising either as the main topic of the video or a segment. None of the products advertised in videos (e.g. chanca piedra, avocado seed tea) had any background research supporting their efficacy. YouTube videos did not include any advertisements.

Misinformation was found in 34% of TikTok videos. These included the claims made in the advertisements in addition to the promotion of home remedies and recipes as methods of preventing and treating kidney stones (e.g. avocado seed tea) or the claims of cure of KS (e.g. limes). Overt misinformation was found in only one YouTube video regarding recipes for curing kidney stones (See supplementary data for misinformation contained in videos).

### Quality rating agreement raters

Kendall’s W was utilised to determine if there was agreement between the 4 raters judgment on each of the quality domains in the TikTok and YouTube videos. Under each category the quality was rated from 1 (very poor) to 5 (very good). Where there was no information included related to each dimension that video was given a ‘0’ rating. The results across platforms illustrate significant agreement across all category ratings (see supplementary data). To account for the fact that many of the TikTok videos contained only one domain of information, e.g. treatment, we recalculated Kendall’s W across both platforms and all domains but containing only the videos which received a rating by at least 1 rater to avoid inflating the level of concordance between raters.

There was statistically significant agreement between raters across all domain categories for both TikTok and YouTube across the 50 videos. On the basis of interpretation criteria (0 = No agreement, 0.10 = Weak agreement, 0.30 = Moderate agreement, 0.60 = Strong agreement, 1 = Perfect agreement) [15] there was strong agreement across all 50 YouTube videos and moderate to strong across TikTok. When ratings were included of only videos including that quality dimension (thus excluding the videos not containing information on e.g. treatment) the level of agreement reduced and in the case of symptoms and epidemiology in TikTok no longer reached significance.

## Overall quality of videos

Just under half (44%) of YouTube videos received a mean score of 3/5 or more whilst 94% of TikTok videos scored 1/5 or less. Only 4 YouTube videos achieved high ratings across all quality categories.

## Differences between YouTube and TikTok

Mean video quality was significantly higher on YouTube than TikTok, (z=-7.378 *p* < 0.001). Apart from the prevention category, YouTube had significantly higher scores than TikTok (general information (z=-6.50 *p* < 0.001), epidemiology (z=-4.79 *p* < 0.001), symptoms/presentation (z=-6.49 *p* < 0.001), treatment (z=-7.24 *p* < 0.001).

There were no significant correlations between video quality (total scores) and their number of views, likes, or comments (p’s > 0.06).

## Discussion

This was the first study to examine the quality of the content of videos about kidney stones available on the most popular social media platforms, currently YouTube and TikTok. The popularity of these platforms for seeking health information was evident in that there were over 46 million views of the videos in the study. Generally, there was considerable variability in the quality of information provided across the domains of general information, epidemiology, symptoms/presentation, treatment, and prevention with only 4 of the videos across the two platforms succeeding in providing good information across all ratings categories.

Ratings of the top 50 KS videos on each platform identified that while there was some variability in the ratings between platforms, overall YouTube had a much higher standard of information quality compared to TikTok. It is likely that this higher rating for YouTube is related to the finding that the majority of YouTube videos (90%) were uploaded by healthcare professionals. While the shorter timeframe of the TikTok videos thus reducing the number of areas of information provided, we note that YouTube also scored higher than TikTok in four out of five categories.

The findings of this study are important for a number of reasons. The current era of personalised medicine highlights the role of the patient in the shared decision-making process and demands that patients are educated about their condition. Previous studies have indicated that individuals with and without kidney stones have a poor understanding of what influences stone formation [16, 17]. Available evidence also suggests that online information available to patients with KS is set at above the recommended reading levels for such information [10], and is not comprehensive [11]. Given this, it becomes increasingly likely that individuals will access resources that are perhaps perceived to be more easily accessible, relatable and understandable such as via social media platforms. We also note that previous studies have shown that viewership of videos can be influenced by factors such as ‘likes’ [18], however, in this study the number of likes was not significantly related to quality highlighting further the importance of patients being directed to material that is both accessible as well as unbiased, accurate and comprehensive. The results of the current study suggest that the most viewed videos across the two most popular social media platforms at the time of this study were not meeting these requirements.

Both platforms contained misinformation which was mostly inaccurate dietary advice, supplements, and the use of exaggerated claims in terms of home remedy effectiveness. While this is commensurate with previous studies exploring online medical misinformation [13, 19, 20], it remains concerning given the popularity of these platforms for medical information. Both platforms have policies in place against content containing misinformation. TikTok explicitly states that health misinformation which may cause negative effects on an individual’s health or may delay seeking medical care for a life-threatening disease is not allowed on the platform. YouTube’s policy is more comprehensive, reporting a long-term plan of fighting misinformation, and considers medical misinformation as information which contradicts local health authorities or the World Health Organization. While both platforms state that content which violates their policies are removed from the platforms once detected and that YouTube further may penalise uploaders with a channel strike (three strikes result in account removal), our study found that over a third of TikTok videos contained misinformation and thus more robust policy violations methods and uploader verification strategies are required to help to ensure less misinformation within content.

### Limitations

As with any study of this nature, there are some limitations. The inclusion of videos only available with English audio or subtitles meant videos available in other languages were excluded from the study, regardless of a video’s position in the search results. While 13 videos were excluded on this basis, it is unlikely that this had any significant influence on the results. Although it is possible that videos beyond the top 100 reviewed may have contained more comprehensive quality content, we decided on the basis of a similar study [19] to review only the top 50 across each platform. We acknowledge that kidney stone information is available on other SoMe. YouTube and TikTok were chosen due to being the two most popular video only platforms with similar search tools available to users. Finally, this study did not set out to examine the suitability of videos for patient consumption, such as the understandability of SoMe videos and as such, we are only able to comment on quality according to AUA criteria [14].

## Conclusions

Although the YouTube and TikTok videos reviewed in the current study showed variability, they were generally of poor quality and were not comprehensive. While YouTube’s quality was rated as better, patients should not be encouraged to access these as a source of reliable information about their kidney stones. There is a clear need for the development of video resources for patients in relation to kidney stones, utilising accurate and unbiased information, and developed alongside patients to ensure the most acceptable and accessible format.

## Electronic supplementary material

Below is the link to the electronic supplementary material.

Supplementary Material 1

## Data Availability

No datasets were generated or analysed during the current study.
