# Supplementary material for: An examination of the quality of kidney stone information on YouTube and TikTok
Source: Urolithiasis. 2025 Feb 25;53(1):40. doi: 10.1007/s00240-025-01713-4 (PMC11861121; doi:10.1007/s00240-025-01713-4)
Supplement: Supplementary file 1 — Supplementary Material 1 [file 240_2025_1713_MOESM1_ESM.docx]

**Supplementary Data Table 1: Kidney Stone Misinformation found in Videos**

| Herbal Remedies   - Chanca Piedra - Avocado seed tea - Cherry stem tea - Cherry stem powder - Marshmallow root - Corn silk - Juniper berries - Uva ursi - Nettle leaf - Black seeds | - Dietary Advice   - Yogurt recipes   - Wrap recipes - Kidney stone curing supplements |
| --- | --- |

**Supplementary Data Table 2. Agreement between raters for each quality domain across platforms for all videos (n=50) and only those contain information related to that quality domain.**

| Quality category | TikTok | YouTube |
| --- | --- | --- |
| General information (n=50)  Scored 1-5 (n) | W=0.47, p<0.001  W=0.37, p=0.048 (n=31) | W=0.70, p<0.001  W=0.69, p<0.001 (n=49) |
| Epidemiology  Scored 1-5 (n) | W=0.74, p<0.001  W=0.5, p=0.14 (n=3) | W=0.68, p<0.001  W=0.56, p<0.001 (n=33) |
| Symptoms  Scored 1-5 (n) | W=0.43, p=0.002  W=0.23, p=0.51 (n=9) | W=0.87, p<0.001  W=0.82, p<0.001 (n=39) |
| Treatment  Scored 1-5 (n) | W=0.60, p<0.001  W=0.43, p=0.04 (n=18) | W=0.87, p<0.001  W=0.84, p<0.001 (n=48) |
| Prevention  Scored 1-5 (n) | W=0.77, p<0.001  W=0.75, p<0.01 (n=49) | W=0.89, p<0.001  W=0.75, p<0.001 (n=31) |
